# GeneGenie: enhancing biomedical question-answering with agentic graphs

**DOI:** 10.1093/bib/bbag430

**Published:** 2026-08-05

**Authors:** Mahmoud Gamal Abdelsalam, Abdulaziz H El-Safty, Amine Zaidi, Tanvir Alam

**Affiliations:** College of Science and Engineering, Hamad Bin Khalifa University, Doha, Qatar; College of Science and Engineering, Hamad Bin Khalifa University, Doha, Qatar; College of Science and Engineering, Hamad Bin Khalifa University, Doha, Qatar; Medical Education, Sidra Medicine, Doha, Qatar; College of Science and Engineering, Hamad Bin Khalifa University, Doha, Qatar

**Keywords:** large language model (LLM), biomedical question answering, retrieval-augmented generation (RAG), agentic graph

## Abstract

Large language models (LLMs) have revolutionized biomedical research, yet they remain prone to hallucinations and struggle with the precise, multi-hop reasoning required for biomedical analysis. To bridge this gap between generative capability of AI model and factual rigor, this article introduces GeneGenie, a model-agnostic, multi-agent framework built upon a directed acyclic graph architecture. Unlike static prompting strategies, GeneGenie implements a deterministic five-node pipeline that orchestrates query planning, intelligent retrieval-augmented generation across curated databases (GenCC, HGNC, and UniProt), and the dynamic execution of bioinformatics tools, including NCBI E-Utilities and local BLAST+. We evaluated the system using the updated 16-module GeneTuring benchmark, comprising 1600 question–answer pairs. The experimental design compared six state-of-the-art models—including GPT-4o, Claude Sonnet 4.5, and Gemini 2.5 Pro—operating in a standalone “Direct Mode” versus the agentic “Graph Mode.” The results demonstrate that the graph-based architecture consistently outperforms single-model baselines across all metrics. Notably, among the six selected LLM models we explored, Gemini 2.5 Pro achieved the highest performance, correctly answering 1158 questions (72.375% accuracy), compared with the best baseline score of only 15.8%. Furthermore, our evaluation utilized an “LLM-as-Judge” semantic assessment, revealing that the agentic approach significantly enhances not only lexical accuracy but also the completeness and factual grounding of responses. While limitations remain in named entity recognition for protein-coding genes, GeneGenie establishes a robust, reproducible paradigm for future biomedical AI systems, proving that tool-augmented orchestration is superior to reliance on raw model scale alone.

## Introduction

Large language models (LLMs) have evolved into indispensable tools that are ubiquitous across diverse domains. Models such as OpenAI GPT [1], Google Gemini Pro [2], and Anthropic Claude [3] have been trained on massive datasets to support a wide array of applications. These capabilities include generating hyper-realistic images and videos, synthesizing software code, and addressing routine inquiries. Moreover, these models produce human-like writing, speeches, and dialects with high fidelity [4]. Recently, LLMs have demonstrated significant promise in biomedical applications [5], particularly in the domain of question-answering (QA) [6]. Advances in instruction-tuned models, retrieval-augmented pipelines, and tool-enabled reasoning have enhanced performance on clinical and research tasks [7–9]. This progress is further bolstered by the extensive availability of biomedical data, such as DNA sequencing and protein coding datasets [10]. These resources enable researchers to leverage LLMs to generate novel insights, thereby accelerating the process of accurate inquiry resolution.

Over the past 6 years, significant advancements have been made in applying LLMs to omics-based research. Pretrained models have demonstrated substantial efficacy in natural language processing (NLP). These architectures generally fall into two primary categories: (i) BERT-like models, such as BioBERT [11], which were pretrained on over 30 million biomedical articles from PubMed, followed by PubMedBERT [12]; and (ii) GPT-like models, developed to address the limited generative capabilities of BERT architectures. Prominent examples include BioGPT [13] and BioMedGPT [14]. More recently, researchers introduced BioMedLM [15], a GPT-style model with 2.7 billion parameters, trained exclusively on PubMed abstracts and full-text articles.

In early 2024, the field experienced a paradigm shift when a team from the NCBI, led by Qiao Jin, introduced GeneGPT, an LLM designed to resolve genome-related queries by accessing NCBI Web APIs [16]. Relying on prompting OpenAI Codex [17], their model achieved an average performance score of 0.83 against GeneTuring, a standard benchmark for genomic QA [18]. While GeneGPT demonstrated the feasibility of prompt-based tool usage, SeqSnap—a GPT-based tool developed as part of the GeneTuring study using OpenAI’s GPT Builder platform [19]—represents the maturation of this concept. SeqSnap transitions the methodology from a static prompt to a robust, production-ready dynamic agent.

Despite these advances, current biomedical QA systems face persistent limitations, including an overreliance on single-model architectures, the generation of plausible yet incorrect outputs (hallucinations), limited generalization across diverse biomedical tasks, and dependence on outdated benchmarks [16, 20]. While retrieval augmentation [21–23], program-generated reasoning [24], and tool-augmented capabilities [25, 26] offer promising solutions, prior work remains predominantly LLM-specific. Currently, a critical gap exists for a model-agnostic, agent-driven system that integrates multi-hop reasoning, domain-specific APIs, and curated genomic knowledge sources while being rigorously evaluated against comprehensive, modern biomedical QA datasets.

The “Graph of Thoughts” framework offers a potential solution [27]. This approach fundamentally challenges the prevailing linear “Chain-of-Thought” paradigm by introducing a graph-based architecture where LLM reasoning steps are modeled as nodes in a directed graph. By facilitating nonlinear pathways—such as branching, backtracking, and aggregating information from distinct agents—models can solve complex tasks with significantly greater efficacy than linear chains. This work provided the theoretical foundation for frameworks such as LangGraph [28]. Recent studies have subsequently applied the LangGraph architecture to biomedical applications [29–31].

Contemporary research is now focusing on the next generation of LLMs to enhance genomic QA. The domain of open-source multi-agent systems for genomics was expanded by the proposal of OpenBioLLM, an extension of GeneGPT presented in [32]. This modular multi-agent framework introduces agent specialization for query generation, tool routing, and response verification, with authors reporting marginal improvements over GeneGPT in 8 out of 12 modules of the original GeneTuring benchmark. However, this work is limited by its reliance on the older 12-module GeneTuring version, a complex graph structure with excessive nodes, and dependence on earlier model architectures such as Qwen 2.5 and Llama 3.1.

Most recently, NVIDIA released a preprint introducing “ToolOrchestra,” a method designed to enhance AI efficiency and capability by training a lightweight, cost-effective model to function as an intelligent tool manager [33]. The objective is to transition from relying on a single, computationally expensive “super-brain” to an orchestrator that intelligently delegates tasks to the optimal expert—whether a web search, a calculator, or a larger model like GPT-5—based on problem complexity. The resulting “Orchestrator-8B” model achieves higher accuracy than GPT-5 on complex reasoning benchmarks, while being 2.5 times more cost-effective. This demonstrates that strategic resource management is superior to relying solely on raw computing power.

Given that this field represents a critical intersection between engineering and medicine, verifying the accuracy of responses is challenging and requires expertise to scrutinize various sources of information. Different benchmarking criteria have been employed to evaluate LLM accuracy, including MedMCQA [34] and the MMLU Medical Genetics exam [35], a specialized version of multitask accuracy testing based on the foundational work reported in [18]. As the primary standard for evaluation in this study, researchers developed GeneTuring in 2023 to assess the precision of LLMs. This repository originally consisted of 600 QA pairs, divided into 12 distinct modules of 50 pairs each [36]. Recently, an updated GeneTuring compilation was published, comprising 16 sections with 100 questions each, resulting in a total of 1600 QA pairs [20]. The creation of the GeneTuring dataset involved extracting high-fidelity data from the National Center for Biotechnology Information (NCBI) and the University of California Santa Cruz (UCSC) Genome Browser. Unlike general biomedical benchmarks (e.g. PubMedQA) that assess reading comprehension, GeneTuring specifically evaluates “Genomic Knowledge” and “Genomic Reasoning.”

These precedents motivate the development of a LangGraph-based agentic pipeline [37] specialized for medical QA. This pipeline incorporates (i) modular LLM interfaces, (ii) retrieval-augmented knowledge sources, (iii) programmatic tool calls to NCBI [38] and other biomedical APIs, and (iv) curated genomic databases. The system will be evaluated on the advanced GeneTuring benchmark (16 modules) [20] to assess accuracy, hallucination reduction, reasoning transparency, and tool-execution robustness. Our aim is to demonstrate that a model-agnostic, tool-augmented framework outperforms single-model approaches. In contrast to the NVIDIA “Orchestrator-8B” generalist framework, which relies on a learned policy for dynamic tool selection, our approach enforces a deterministic workflow where specific tools are explicitly assigned to distinct reasoning steps. Consequently, our model leverages a structured graph-based reasoning architecture to ensure strict adherence to domain-specific genomics. It will provide a scalable, evidence-based architecture for biomedical QA and benchmark future biomedical AI systems.

The remainder of this article is organized as follows. Section GeneGenie system architecture details the system architecture, describing the LangGraph-based orchestration, the five-stage reasoning pipeline, and the integration of retrieval-augmented generation (RAG) and bioinformatics tools. Section Evaluation and results presents the comprehensive evaluation framework and experimental results, contrasting the performance of graph mode against direct mode across varying model families and GeneTuring modules. Section Limitations of GeneGenie provides a critical analysis of the system’s limitations, specifically addressing challenges in task disambiguation and entity recognition. Finally, Section Conclusion and future directions concludes the study and outlines future directions for enhancing node specialization and self-validation loops in biomedical agents.

## GeneGenie system architecture

**Figure 1.**
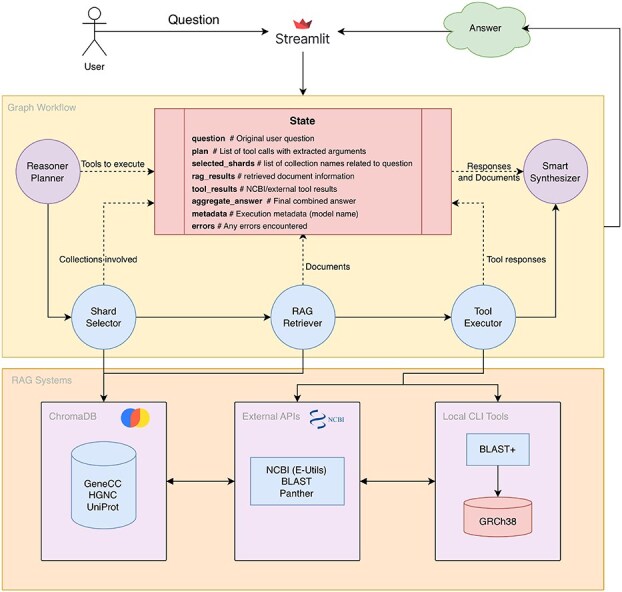
GeneGenie architecture and query pipeline A Streamlit interface initiates a state-driven Graph Workflow where five specialized nodes iteratively update a shared State. Execution strategies (Reasoner Planner) drive localized semantic retrieval from ChromaDB vector stores (Shard Selector and RAG Retriever) alongside bioinformatic querying via External APIs and local BLAST+ tools against GRCh38 (Tool Executor). The Smart Synthesizer compiles the final multi-source response. Solid arrows map data flow; dashed arrows indicate state interactions.

GeneGenie implements a multi-agent biomedical QA system built upon a LangGraph-based orchestration framework that integrates RAG, specialized bioinformatics tools, and multiple LLM providers. The system architecture comprises three primary layers: (i) a user interface layer implemented in Streamlit, (ii) a LangGraph orchestration layer containing five specialized agent nodes, and (iii) a knowledge and tool integration layer encompassing RAG systems, external APIs, and local computational resources as depicted in Fig. 1.

The orchestration layer operates as a directed acyclic graph where each node performs a discrete function while maintaining a shared state object. This state-based design enables information flow across nodes while preserving intermediate results for downstream synthesis. The system supports model-agnostic LLM integration through a unified provider abstraction layer, enabling seamless switching between OpenAI GPT-4, Anthropic Claude 4.5, and Google Gemini models without architectural modifications.

### Multi-agent workflow pipeline

The system implements a deterministic five-stage pipeline shown in Fig. 1, where each agent node contributes specialized capabilities.

#### Stage 1: reasoner-planner node

This node performs unified query analysis and execution planning through a single LLM invocation. The reasoner component analyzes query complexity, identifies required information sources, and determines the optimal tool execution sequence. The planner component generates a structured execution plan represented as a JSON array of tool calls, each containing: (i) a sequential step identifier, (ii) the tool name, (iii) extracted arguments parsed directly from the natural language query, and (iv) a reasoning justification.

#### Stage 2: shard selector node

To optimize retrieval efficiency, this node implements intelligent RAG collection filtering based on keyword analysis. The node extracts domain-specific terms from the query (e.g. “gene,” “protein,” and “disease”) and maps them to relevant ChromaDB collections. The selection logic follows a predefined mapping:

- Gene-related queries (keywords: “gene,” gene symbols) $\rightarrow$  gene_info, gencc
- Protein queries (keywords: “protein,” protein names) $\rightarrow$  protein_info
- Disease association queries (keywords: “disease,” pathology terms) $\rightarrow$  gencc

#### Stage 3: RAG retriever node

The RAG system queries the selected ChromaDB collections using semantic similarity search based on OpenAI’s text-embedding-3-small model (1536D embeddings). Each collection is queried independently with a configurable n_results parameter (default: three documents per collection). Retrieved documents are ranked by cosine similarity and aggregated with metadata, including source collection, similarity scores, and document identifiers.

The RAG knowledge base comprises three curated collections:

1. **GenCC collection**: High-confidence gene–disease associations from the Gene Curation Coalition, filtered for “Definitive” and “Strong” evidence levels ($\sim$2500 documents). The dataset corresponds to the GenCC dump downloaded on 29 September 2025 and was used without further modification.
2. **Gene info collection**: Approved gene nomenclature from HGNC, including gene symbols, aliases, and genomic locations ($\sim$19 000 documents). This dataset was downloaded on 18 November 2025 and used without further modification.
3. **Protein info collection**: Protein functional annotations from UniProt, including protein names, functions, and gene cross-references ($\sim$20 000 documents; Release 2025_03, June 2025) and used without further modification.

We have highlighted the format of embedded chunks in Supplementary Material S1.

#### Stage 4: tool executor node

This node executes the planned tool sequence with comprehensive error handling and rate limiting. The system integrates seven specialized bioinformatics tools:

1. search_gene: Queries NCBI Gene database via E-utilities (ESearch + ESummary) with organism filtering
2. search_dbsnp: Retrieves SNP pathogenicity information from dbSNP database
3. search_tf_regulation: Determines transcription factor regulatory modes (activation/repression)
4. fetch_genomic_sequence: Extracts DNA sequences from chromosomal coordinates using NCBI EFetch
5. translate_sequence: Performs six-frame translation using Biopython’s translation engine
6. run_blast: Unified BLAST interface with organism-aware routing (detailed below)
7. enrich_pathways: Conducts pathway enrichment analysis via PantherDB API.

Tool execution follows a sequential order with dependency tracking, where results from earlier tools may inform arguments for subsequent tools. Failed tool executions are logged but do not terminate the pipeline; instead, errors are captured in the state object for synthesis consideration. Two main tools integrations done here:

**(1) NCBI API integration.** All NCBI tools implement exponential backoff retry logic (maximum three attempts) to handle transient network failures and rate limiting. The system respects NCBI’s rate limits (10 requests/second with API key, 3 requests/second without) through intelligent request throttling.

**(2) BLAST integration.** The system implements a novel dual-mode BLAST architecture:

- **Local BLAST**: For human genome queries, the system executes BLAST+ CLI against a local hg38 reference database (30 GB), achieving 1–3 s query times (100$\times$ faster than remote BLAST). The local BLAST+ database was built with 455 sequences (chromosomes, alternate contigs, and patches) totaling 3 209 286 105 bases. The database was formatted with BLAST+ version 5 using the BLAST 2.17.0 package (build date: 1 July 2025; database creation date: 24 November 2025).
- **Remote BLAST**: For nonhuman organisms, the system submits queries to NCBI BLAST API with database selection (nt) and retrieves results via XML parsing.

The organism-aware routing logic automatically detects organism mentions in queries (e.g. “human,” “mouse,” and“zebrafish”) and selects the appropriate BLAST mode. This hybrid approach optimizes performance for the most common use case (human genomics) while maintaining flexibility for comparative genomics.

#### Stage 5: smart synthesizer node

The final node performs evidence integration and response generation through LLM-based synthesis with strict formatting enforcement. The synthesizer receives: (i) RAG retrieval results with similarity scores, (ii) tool execution outputs with metadata, and (iii) the original question.

The synthesis process follows a priority hierarchy where RAG results override tool results for gene–disease associations, given the high-confidence curation of the GenCC database. The LLM is prompted with explicit formatting rules:

- Genomic coordinates: chr[N—X—Y]:[start]-[end] format
- Protein translations: Amino acid sequence only, no metadata
- Gene/protein names: Only entities explicitly mentioned in source documents
- Regulatory modes: Single-word responses (Activation/Repression)
- Species names: Common names preferred over Latin nomenclature

Meta-prompting techniques enforce direct answering without preamble phrases (e.g. “According to,” “The results show”).

### State management and data flow

The system employs a strongly typed state schema (BioState) that persists across all nodes, implemented using Python dictionaries with Pydantic validation. The state object contains:

- **Input fields**: User question, timestamp, session metadata
- **Planning outputs**: Structured tool execution plan with arguments
- **Retrieval outputs**: Selected shard identifiers, RAG documents with metadata
- **Tool outputs**: Execution results, error logs, latency metrics
- **Synthesis outputs**: Final answer, extracted citations, confidence scores
- **Evaluation metrics**: ROUGE scores, LLM-judge ratings

This centralized state management enables full observability through LangSmith tracing, where each node transition, LLM invocation, and tool call is recorded for debugging and performance analysis.

### Multi-large language models provider support

The system abstracts LLM providers through a unified LLMProvider interface that normalizes API calls across OpenAI, Anthropic, and Google platforms. All LLM invocations use deterministic sampling (temperature=0.0) to ensure reproducible outputs. The provider selection is configurable at runtime, enabling comparative evaluation across models without code modifications.

### Evaluation framework

We developed a comprehensive evaluation framework supporting two operational modes:

1. **Graph mode**: Full pipeline evaluation with RAG retrieval and tool execution enabled, representing the complete GeneGenie system.
2. **Direct mode**: Baseline evaluation where the LLM receives only the question without access to RAG or tools, enabling assessment of tool-augmentation benefits.

The framework processes evaluation datasets in CSV format (columns: Question, Gold standard, and Module) and computes multiple metrics:

1. **ROUGE metrics**: ROUGE-1, ROUGE-2, and ROUGE-L scores measuring lexical overlap between generated answers and gold standard references.
2. **LLM-as-judge evaluation**: GPT-4-based assessment of accuracy, completeness, relevance, and citation quality on a 0–10 scale.
3. **Exact match evaluation**: Measurement of perfect correspondence between predicted outputs and gold standard answers of GeneTuring benchmark.
4. **Performance metrics**: Correct count, latency, and successful request count for assessing accuracy and system efficiency.

## Evaluation and results

To comprehensively assess the performance of the multi-agent architecture, we conducted systematic experiments across multiple dimensions: model selection, operational modes, and evaluation metrics. We evaluated six state-of-the-art LLMs from three major providers: Anthropic (Claude Sonnet 4.5, Claude Opus 4), OpenAI (GPT-4o, GPT-4o-mini), and Google (Gemini 2.5 Flash, Gemini 2.5 Pro). Each model was tested in two operational modes: *Graph Mode*, representing the complete graph-based system with RAG retrieval and tool execution, and *Direct Mode*, serving as an LLM-only baseline without access to external knowledge sources or computational tools. Our evaluation framework employed the GeneTuring benchmark dataset comprising 1600 QA pairs distributed. We present the structure of prompts used in Supplementary Material S2.

The following subsections present our findings organized by evaluation dimension, demonstrating the efficacy of the tool-augmented architecture compared with direct LLM invocation and revealing model-specific performance characteristics across genomic reasoning tasks.

### Correct answer comparison: graph versus direct mode

**Figure 2.**
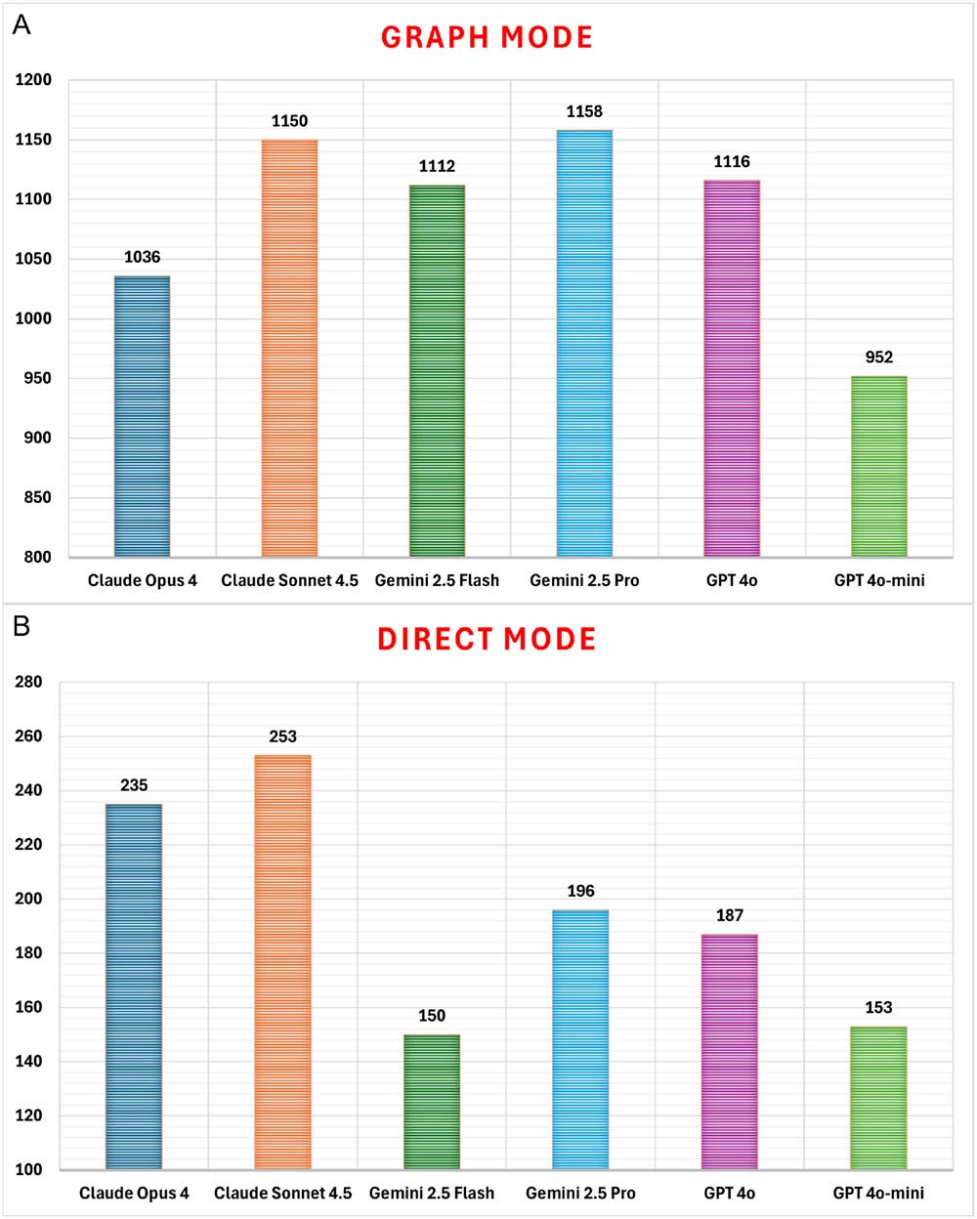
Comparison of correct answer counts between (A) graph mode (tool-augmented) and (B) direct mode (LLM-only baseline) across six evaluated models. Graph mode consistently outperforms direct mode across all model architectures, demonstrating the benefit of RAG retrieval and specialized bioinformatics tool integration.

To evaluate the overall efficacy of the tool-augmented architecture, we computed exact match accuracy by comparing model-generated answers against gold-standard references across all 1600 GeneTuring questions, irrespective of module categorization. Figure 2 shows the aggregate correct answer counts for each model in both operational modes.

The results demonstrate a consistent and substantial performance advantage for graph mode across all evaluated models. Every model operating in graph mode surpassed its corresponding direct mode baseline, validating the hypothesis that integration of RAG retrieval and specialized bioinformatics tools significantly enhances answer correctness. Notably, five of the six models in graph mode exceeded the 1000 correct answers threshold (62.5% accuracy), with Gemini 2.5 Pro achieving the highest performance at 1158 correct answers (72.375% accuracy). The sole exception was GPT-4o-mini, which nonetheless showed marked improvement in graph mode (952 correct) compared with direct mode.

In contrast, direct mode performance remained considerably lower across all models, with the best-performing baseline (Claude Sonnet 4.5) achieving only 253 correct answers (15.85% accuracy). This performance gap—ranging improvement depending on the model, underscores the critical role of external knowledge sources and computational tools in resolving complex genomic queries that extend beyond the parametric knowledge encoded in pretrained language models.

### Module-level performance analysis

**Figure 3.**
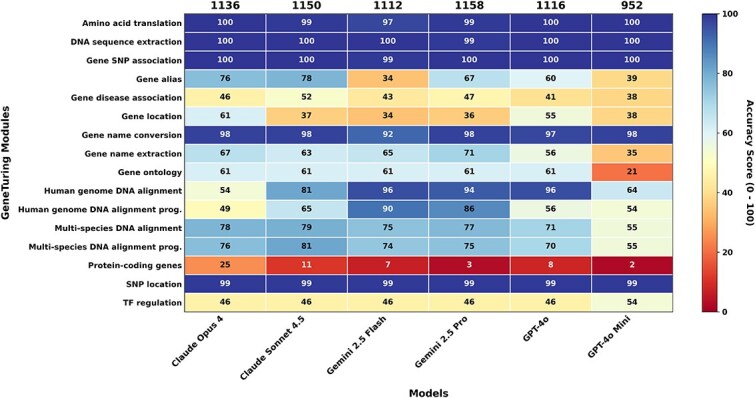
Heatmap showing per-module correct answer rates for each model in graph mode across GeneTuring’s 16 evaluation categories. Columns represent individual models; rows represent genomic task modules. Accuracy scores (0–100) are visualized using a divergent color scale, ranging from lowest to highest performance scale. This visualization reveals model-specific strengths and weaknesses across different genomic reasoning tasks.

To identify task-specific strengths and weaknesses of the graph mode architecture, we analyzed correct answer rates across GeneTuring’s 16 specialized modules, as visualized in Fig. 3. This granular analysis reveals substantial performance heterogeneity across genomic reasoning task categories, with certain modules demonstrating near-universal model proficiency while others expose systematic challenges. For compatibility with earlier baselines, we additionally evaluate the graph mode architecture on the original GeneTuring dataset, comprising 12 modules with 50 questions each. Results are provided in Supplementary Material S3.

#### High-performance modules

Several tasks achieved consistently strong performance across all evaluated models. Amino acid translation queries exhibited the highest accuracy, with all models exceeding 99% correctness, likely attributable to the deterministic nature of genetic code translation and reliable Biopython implementation in the tool executor. Similarly, gene-SNP association tasks demonstrated robust performance (median accuracy: 100%), benefiting from the comprehensive dbSNP API coverage.

#### Low-performance modules

The protein-coding gene identification task exhibited the poorest performance across all models (median accuracy: 8%), representing a critical limitation of the current system. This failure mode stems from ambiguities in NCBI Gene database annotations and insufficient prompt engineering to handle edge cases where genes possess both coding and non-coding isoforms. Detailed analysis of this limitation and proposed mitigation strategies are discussed in Section Limitations of GeneGenie.

Figure 3 illustrates these patterns through color-coded gradient to measure the performance, revealing that while graph mode architecture provides substantial benefits across most genomic reasoning tasks, targeted improvements in tool integration and prompt design remain necessary for achieving uniformly high performance across all GeneTuring categories.

### Lexical similarity: ROUGE score analysis

To assess lexical overlap between model-generated answers and gold standard references, we computed ROUGE (Recall-Oriented Understudy for Gisting Evaluation) metrics across three variants: ROUGE-1, ROUGE-2, and ROUGE-L. These complementary metrics quantify different aspects of textual similarity:

- **ROUGE-1**: Measures unigram (single-word) overlap between prediction and reference, capturing vocabulary similarity regardless of word order.
- **ROUGE-2**: Measures bigram (two-word sequence) overlap, assessing preservation of local word ordering and phrase structure.
- **ROUGE-L**: Measures longest common subsequence (LCS), quantifying the longest in-order matching word sequence, which captures overall sentence structure similarity while allowing for insertions.

**Figure 4.**
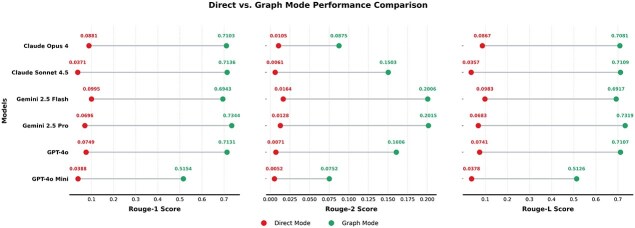
ROUGE score comparison (ROUGE-1, ROUGE-2, and ROUGE-L) between graph mode and direct mode for all evaluated models. Graph mode generally achieves higher ROUGE scores, indicating improved alignment with reference answers through integration of curated databases and computational tools.

Figure 4 presents ROUGE score distributions across all models and operational modes. Several notable patterns emerge from this analysis.

A substantial performance gap exists between graph mode and direct mode across all ROUGE variants for all evaluated models, with graph mode consistently achieving higher scores. This indicates that tool-augmented responses exhibit greater lexical fidelity to reference answers, likely due to the retrieval of terminology-rich database entries and structured tool outputs that match gold-standard formatting conventions.

ROUGE-2 scores are markedly lower than ROUGE-1 across all conditions, with typical reductions of 60%–70%. This degradation reflects the challenge of preserving exact two-word phrases in genomic answers, where models may use synonymous expressions or reorder technical terms while maintaining semantic correctness. The reduced bigram overlap suggests that while models capture relevant vocabulary (ROUGE-1), they struggle to replicate precise phrasing patterns.

ROUGE-1 scores exhibit relative consistency across most models (ranging 0.69–0.71 in graph mode), with the notable exception of GPT-4o-mini, which demonstrates substantially lower performance (0.51). This outlier behavior aligns with GPT-4o-mini’s reduced correct answer count (Fig. 2), suggesting that the smaller model architecture produces answers with both lower factual accuracy and reduced lexical similarity to references.

The near-equivalence of ROUGE-L and ROUGE-1 scores provides diagnostic insight into answer structure. This pattern indicates that models successfully incorporate correct terminology (high unigram overlap) and maintain reasonable sentence-level ordering (high LCS overlap), but introduce extraneous words or phrases that prevent exact matching. These additional tokens, such as preamble phrases, explanatory clauses, or formatting variations, preserve the core factual content while degrading exact match accuracy. This observation suggests that post-processing or stricter output formatting constraints could substantially improve exact match rates without requiring architectural changes.

Collectively, these ROUGE analyses confirm that graph mode enhances lexical fidelity through grounded knowledge retrieval, while revealing opportunities for output refinement to better align generated answers with reference formatting conventions.

### Semantic quality assessment: large language models-as-judge evaluation

To complement lexical similarity metrics with human-aligned semantic evaluation, we employed two different LLM models, GPT-4o and Gemini 2.5 flash, as an automated judge to assess answer quality. Each dimension was rated on a 0–10 scale, with higher scores indicating superior performance. This evaluation methodology captures semantic correctness and evidential rigor that pure lexical metrics may overlook, such as paraphrased but accurate answers or well-supported claims.

**Figure 5.**
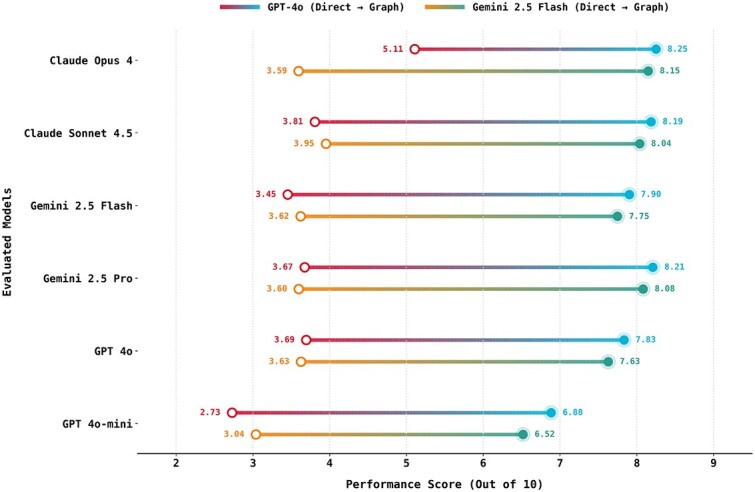
LLM-as-judge comparative evaluation of model performance for both graph mode and direct mode. Scores range from 0 to 10, with higher values indicating superior performance. For each evaluated model, paired dumbbell lines track the performance leap from direct mode (empty circles) to the multi-agent graph mode (filled circles).

Figure 5 depicts the aggregate LLM-judge scores across all evaluated models in direct versus graph mode, as assessed by two independent LLM judges (GPT-4o and Gemini 2.5 Flash). The results reveal that transitioning from direct to graph mode results in a performance leap (an average absolute gain of 4+ points on a 10-point scale). This substantially matches the differences observed in ROUGE metrics. Notably, neither judge demonstrated a significant “self-bias” (favoring their own underlying model). For instance, the GPT-4o judge rated Claude Opus 4 and Gemini 2.5 Pro higher than itself in graph mode. Similarly, the Gemini 2.5 Flash judge rated Claude Opus 4 higher than itself. This lack of bias strengthens the credibility of the evaluation methodology. There is a slight divergence in how the judges viewed Claude Opus 4 in direct mode. GPT-4o gave it a 5.10, while Gemini gave it a more conservative 3.59. However, this discrepancy vanishes once the models enter graph mode, suggesting the orchestration layer standardizes the quality of output to a point where judge-specific subjectivity is minimized.

These LLM-judge evaluations provide strong evidence that the multi-agent architecture enhances not merely lexical similarity but fundamental semantic quality, producing answers that are factually accurate and comprehensive. The dramatic performance differential between modes validates the necessity of tool-augmented approaches for production biomedical QA systems where answer quality directly impacts clinical and research decisions.

### Computational efficiency: inference time analysis

While accuracy and semantic quality constitute primary performance objectives, computational efficiency remains critical for production deployment of biomedical QA systems. To assess the practical viability of the multi-agent architecture, we measured end-to-end inference time for all graph mode queries, capturing the complete pipeline latency from question submission through final answer synthesis. These measurements encompass all processing stages: reasoner-planner analysis, shard selection, RAG retrieval, tool execution (including NCBI API calls and BLAST queries), and LLM-based synthesis.

**Figure 6.**
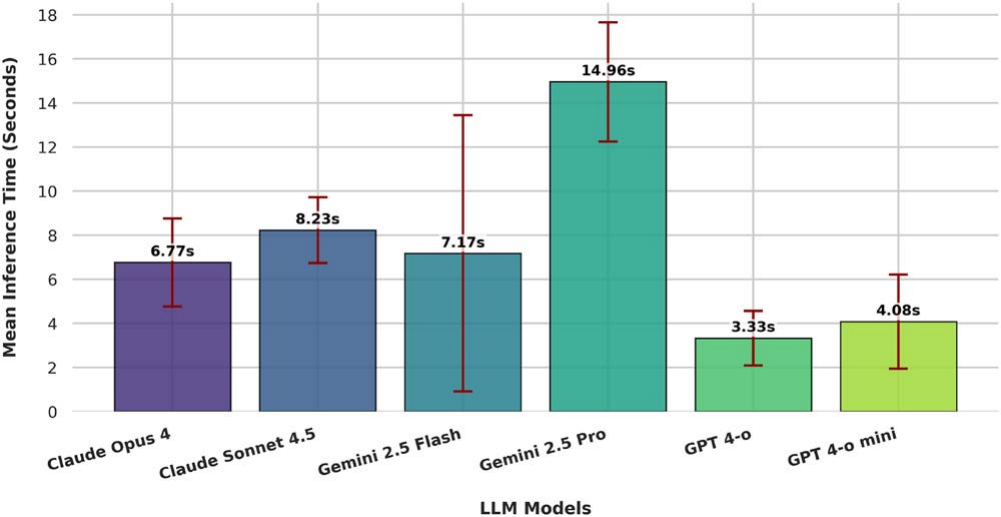
Mean end-to-end inference time for graph mode queries across evaluated LLM models. Bar heights represent average response times in seconds, with error bars indicating performance variability.

Figure 6 presents the distribution of inference times across all evaluated models in graph mode as stated in Table 1. The results reveal mean times range from 3.33s (GPT 4o) to 14.96s (Gemini 2.5 Pro). These results demonstrate efficient, production-ready performance despite the computational overhead of multi-agent orchestration, RAG retrieval, and external tool execution. Variation across models reflects differences in provider API latency and model-specific reasoning speeds.

**Table 1:** Mean and standard deviation (SD) of inference time across different LLM models.

Model	Mean time (s)	SD
Claude Opus 4	6.77	2.00
Claude Sonnet 4.5	8.23	1.50
Gemini 2.5 Flash	7.17	6.27
Gemini 2.5 Pro	14.96	2.70
GPT 4o	3.33	1.23
GPT 4o mini	4.08	2.13

Table 2 presents the performance of the proposed work, GeneGenie, in comparison with four other models reported in the literature, using the GeneTuring benchmark. The comparison includes SeqSnap [20], NanoBioAgent [39], GenomAgent, and GeneGPT-Slim [16]. The latter three were originally evaluated on the earlier GeneTuring version (12 modules), whereas our model and SeqSnap were evaluated on the updated GeneTuring 16-module benchmark. This difference in benchmark coverage explains the presence of NA values across several rows. Gene–SNP association achieved perfect accuracy for all evaluated models. In contrast gene ontology, transcription factor regulation, and the programming-based alignment modules revealed clearer performance differences. GeneGenie demonstrated better accuracy compared with SeqSnap in gene ontology, human genome DNA alignment programming, multispecies DNA alignment programming, and amino acid translation.

**Table 2:** Comparison of different models in the literature utilizing GeneTuring benchmark.

GeneTuring module	Gene genie^a^ (this work)	SeqSnap^b^ [20]	NanoBioAgent^c^ [39]	GenomAgent^c^ [40]	GeneGPT Slim^c^ [16]
**Amino acid translation**	**0.99**	0.11	NA	NA	NA
**DNA sequence extraction**	0.99	**1**	NA	NA	NA
**Gene – SNP association**	**1**	**1**	**1**	**1**	**1**
**Gene alias**	0.67	0.26	**0.98**	0.96	0.84
**Gene-disease association**	0.47	0.57	**0.93**	0.76	0.66
**Gene location**	0.36	0.36	**1**	0.96	0.66
**Gene name conversion**	0.98	0	1	1	1
**Gene name extraction**	0.71	**0.83**	NA	NA	NA
**Gene ontology**	**0.61**	0.12	NA	NA	NA
**Human genome DNA alignment**	**0.94**	0.92	0.93	0.82	0.44
**Human genome DNA alignment programming**	**0.86**	0.19	NA	NA	NA
**Multispecies DNA alignment**	0.77	0.85	**1**	0.88	0.88
**Multispecies DNA alignment programming**	**0.75**	0.55	NA	NA	NA
**Protein coding genes**	0.03	**0.69**	1	1	1
**SNP location**	0.99	**1**	**1**	0.98	0.98
**Transcription factor regulation**	0.46	**0.58**	NA	NA	NA

NA: This module was not incorporated in the model. $^{\mathrm{a}}$(Graph mode) exact answers using Gemini Pro 2.5. $^{\mathrm{b}}$Exact and partially exact answers. $^{{\mathrm{c}}}$Old GeneTuring Version (50 Questions/module). Bold values indicate the highest performing model on the module.

## Limitations of GeneGenie

While graph-augmented retrieval substantially improves results over LLM-only baselines, our analysis on the GeneTuring benchmark reveals several systematic limitations related to task comprehension, retrieval reliability, entity recognition, and pipeline robustness.

### Task disambiguation and prompt sensitivity

GeneGenie occasionally misinterprets task intent when structurally similar tasks require different reasoning objectives. For example, in gene alias resolution tasks, the system sometimes extracts gene symbols directly from the query rather than reporting the canonical symbol retrieved by tools. This reflects insufficient task-specific synthesis instructions and reliance on generic prompts. More explicit module-level synthesis guidance would improve task alignment.

### Retrieval precision and entity disambiguation

Gene–disease association tasks remain sensitive to retrieval quality. The GenCC collection is currently indexed as a single shard, reducing precision for phenotype-driven queries. Additionally, the system occasionally conflates gene and protein terminology, leading to incorrect associations. Improved collection sharding, metadata indexing, and gene–protein disambiguation during retrieval ranking would mitigate these errors.

### Tool reliability and execution errors

Performance is also constrained by tool execution failures. External APIs such as NCBI Gene may return incorrect or ambiguous results when queries are not sufficiently specific. Similarly, smaller models occasionally fail to correctly extract long DNA sequences for downstream alignment tools. These failures highlight the importance of robust query formulation, validation, and tool-input verification.

### Output formatting constraints

In some cases, the system produces scientifically correct but benchmark-incompatible outputs, such as using taxonomic organism names instead of common names. While factually accurate, such formatting discrepancies reduce exact-match evaluation scores. Post-processing normalization or more flexible evaluation criteria would better reflect semantic correctness.

### Named entity recognition limitations

The most significant limitation involves extracting gene and protein names directly from free text. The current system relies heavily on database-based validation, which can lead to false positives through contextual hallucination and false negatives when encountering nonstandard or previously unseen entity names. Integrating specialized biomedical named entity recognition (NER) models would substantially improve entity extraction accuracy.

### Error propagation across pipeline stages

Errors in early stages, particularly entity extraction, propagate to downstream tasks such as gene classification, resulting in incorrect or incomplete predictions. This reflects a broader architectural limitation: the absence of intermediate validation and uncertainty handling. Incorporating confidence estimation and validation checkpoints would improve overall system robustness.

### Single-node evaluation

Comprehensive ablation study of the multi-agent pipeline was not conducted due to computational budget constraints. While the tight coupling of the five nodes imposes an inherent architectural dependency, where each node relying on the correct output of its predecessor, a more resource-intensive evaluation could explore lightweight proxy experiments or partial pipeline variants to further isolate the contribution of individual node components. We leave this as a direction for future work.

## Conclusion and future directions

This work presents GeneGenie, a multi-agent biomedical QA system that integrates LLM reasoning with RAG and specialized bioinformatics tools using a LangGraph-based orchestration framework. Evaluation on the GeneTuring benchmark across six state-of-the-art LLMs demonstrates that tool-augmented architectures consistently outperform LLM-only baselines. graph mode achieved higher accuracy than direct mode across all evaluated models, confirming that performance gains arise from structured tool integration rather than inherent LLM capability alone. These improvements were observed across model families and sizes, demonstrating the model-agnostic nature of the architecture and its adaptability to evolving LLM ecosystems. Beyond performance gains, this work highlights the importance of structured reasoning, tool orchestration, and external knowledge grounding for reliable biomedical QA.

Despite these advances, several opportunities exist for further improvement. First, integrating specialized biomedical NER models would substantially improve gene and protein extraction from free text, reducing hallucinations and missed entities, particularly for novel or nonstandard biological designations. Second, architectural refinements such as separating reasoning and planning into distinct components and introducing intermediate validation nodes would improve task alignment and reduce error propagation. Third, incorporating self-validation and confidence estimation mechanisms would enable the system to detect and correct uncertain predictions. Finally, future work should explore improved evaluation strategies that account for semantic equivalence rather than strict lexical matching, providing a more accurate assessment of biomedical correctness.

Collectively, these directions would enhance the robustness, accuracy, and scalability of tool-augmented biomedical AI systems, supporting their application in real-world genomic analysis and scientific discovery workflows.

**Key Points**

- Orchestration quality, not base model capability, dominates end-to-end accuracy: under graph mode the six evaluated large language models (LLMs) converge to a narrow band of 952–1158 correct answers, whereas the best any model achieves unaided is 253. The scaffolding, rather than model selection, is therefore the more productive engineering target.
- Benefits from tool augmentation are strongly task-dependent rather than uniform. Modules backed by deterministic computation or well-covered APIs approach ceiling performance (amino acid translation exceeds 99% for every model; gene–SNP association reaches a median of 100%), while protein-coding gene identification collapses to a median of 8% because of annotation ambiguity in the underlying databases.
- A substantial share of the remaining error is presentational rather than inferential: ROUGE-L tracks ROUGE-1 closely while ROUGE-2 falls by 60%–70%, indicating that answers carry the correct terminology and ordering but add extraneous preamble. Output normalization could therefore raise exact-match scores without any architectural change.
- Accuracy and latency are decoupled, which matters for interactive deployment: mean end-to-end response time spans 3.3 s (GPT-4o) to 15.0 s (Gemini 2.5 Pro), making the most accurate configuration $\sim$4.5 times slower than the fastest competitive one.
- Dual independent LLM judges (GPT-4o and Gemini 2.5 Flash) exhibited no self-preference, each scoring rival models above its own. Their disagreement on unaided answers (5.10 versus 3.59 for Claude Opus 4) vanished under graph mode, suggesting the orchestration layer standardizes output quality enough to suppress evaluator subjectivity.

## Supplementary Material

Supplementary_bbag430

## Data Availability

Data used in this study are publicly accessible at: https://github.com/Winnie09/GeneTuring/tree/main/GeneGPT. Source code is available at: https://github.com/mahmoudgamal0/GeneGenie.

## References

[1] OpenAI . GPT-4: Advanced Conversational AI 2023. https://openai.com/research/gpt-4 (12 December 2025, date last accessed).

[2] Google Cloud . Gemini 2.5 pro: Advanced Reasoning Model on Vertex AI 2025. https://docs.cloud.google.com/vertex-ai/generative-ai/docs/models/gemini/2-5-pro (12 December 2025, date last accessed).

[3] Anthropic . Introducing Claude Sonnet 4.5 2025. https://www.anthropic.com/news/claude-sonnet-4-5 (12 December 2025, date last accessed).

[4] Song Y, Chen H, Lian J et al. GOAT-TTS: expressive and realistic speech generation via a dual-branch LLM. arXiv preprint arXiv:250412339. 2025.

[5] Nagarajan R, Klotzman V, Kondo M et al. Taxonomy portraits: deciphering the hierarchical relationships of medical large language models. *JMIR Med Inform* 2025;13:e72918. 41060040 10.2196/72918 PMC12505401

[6] Singhal K, Tu T, Gottweis J et al. Toward expert-level medical question answering with large language models. *Nat Med* 2025;31:943–50. 39779926 10.1038/s41591-024-03423-7 PMC11922739

[7] Singhal K, Azizi S, Tu T et al. Large language models encode clinical knowledge. *Nature* 2023;620:172–80. 37438534 10.1038/s41586-023-06291-2 PMC10396962

[8] Liévin V, Hother CE, Motzfeldt AG et al. Can large language models reason about medical questions? *Patterns* 2024;5:100943. 10.1016/j.patter.2024.100943 PMC10935498 38487804

[9] Nori H, King N, McKinney SM, et al. Capabilities of GPT-4 on medical challenge problems. arXiv preprint arXiv:230313375. 2023.

[10] Camacho C, Coulouris G, Avagyan V et al. BLAST+: architecture and applications. *BMC Bioinformatics* 2009;10:421. 20003500 10.1186/1471-2105-10-421 PMC2803857

[11] Lee J, Yoon W, Kim S et al. BioBERT: a pre-trained biomedical language representation model for biomedical text mining. *Bioinformatics* 2020;36:1234–40. 31501885 10.1093/bioinformatics/btz682 PMC7703786

[12] Gu Y, Tinn R, Cheng H et al. Domain-specific language model pretraining for biomedical natural language processing. *ACM Transactions on Computing for Healthcare (HEALTH)* 2021; 3:1–23.

[13] Luo R, Sun L, Xia Y et al. BioGPT: generative pre-trained transformer for biomedical text generation and mining. *Brief Bioinform* 2022;23:bbac409. 36156661 10.1093/bib/bbac409

[14] Luo Y, Zhang J, Fan S, et al. BioMedGPT: open multimodal generative pre-trained transformer for biomedicine. arXiv preprint arXiv:230809442. 2023.

[15] Bolton E, Venigalla A, Yasunaga M, et al. BioMedLM: a 2.7 b parameter language model trained on biomedical text. arXiv preprint arXiv:240318421. 2024.

[16] Jin Q, Yang Y, Chen Q et al. GeneGPT: augmenting large language models with domain tools for improved access to biomedical information. *Bioinformatics* 2024;40:btae075. 38341654 10.1093/bioinformatics/btae075 PMC10904143

[17] OpenAI . Codex: One Agent for Everywhere you Code 2025. https://openai.com/codex/ (12 December 2025, date last accessed).

[18] Hendrycks D, Burns C, Basart S, et al. Measuring massive multitask language understanding. arXiv preprint arXiv:200903300. 2020.

[19] XinyiShang XL, ZhichengJi WH. SeqSnap GPT-Based Tool for Analyzing Genetic Sequences Using NCBI Tools and Running BLAST Alignments 2025. https://chatgpt.com/g/g-67c52efdc210819190a9532f264ec9c0-seqsnap (12 December 2025, date last accessed).

[20] Shang X, Liao X, Ji Z et al. Benchmarking large language models for genomic knowledge with GeneTuring. *Brief Bioinform* 2025;26:bbaf492. 40984701 10.1093/bib/bbaf492 PMC12454257

[21] Guu K, Lee K, Tung Z et al. Retrieval augmented language model pre-training. In: Proceedings of the 37th *International Conference on Machine Learning*, pp. 3929–38. PMLR, 2020.

[22] Lewis P, Perez E, Piktus A et al. Retrieval-augmented generation for knowledge-intensive NLP tasks. *Adv Neural Inf Proces Syst* 2020;33:9459–74.

[23] Borgeaud S, Mensch A, Hoffmann J et al. Improving language models by retrieving from trillions of tokens. In: *International Conference on Machine Learning*, pp. 2206–40. PMLR, 2022.

[24] Gao L, Madaan A, Zhou S et al. PAL: program-aided language models. In: *Proceedings of the 40th International Conference on Machine Learning*, pp. 10764–99. PMLR, 2023.

[25] Parisi A, Zhao Y, Fiedel N. TALM: tool augmented language models. arXiv preprint arXiv:220512255. 2022.

[26] Schick T, Dwivedi-Yu J, Dessì R et al. Toolformer: language models can teach themselves to use tools. *Adv Neural Inf Proces Syst* 2023;36:68539–51.

[27] Besta M, Blach N, Kubicek A et al. Graph of thoughts: solving elaborate problems with large language models. In: Proceedings of the AAAI conference on artificial intelligence, Vol. 38, pp. 17682–90, 2024. Available from: 10.1609/aaai.v38i16.29720

[28] Wang J, Duan Z. Agent AI with LangGraph: a modular framework for enhancing machine translation using large language models. arXiv preprint arXiv:241203801. 2024.

[29] Wehling L, Singh G, Mulyadi AW et al. Talk2Biomodels: AI agent-based open-source LLM initiative for kinetic biological models. *BMC Bioinformatics* 2025;26:276. 41254502 10.1186/s12859-025-06310-1 PMC12625589

[30] Liu W, Li J, Tang Y et al. DrBioRight 2.0: an LLM-powered bioinformatics chatbot for large-scale cancer functional proteomics analysis. *Nat Commun* 2025;16:2256. 40050282 10.1038/s41467-025-57430-4 PMC11885830

[31] Liu S, Xing H, Han M et al. BioMedTools: a language model-powered community for biomedical computational tools. *bioRxiv* 2025;2025–05.

[32] Chen H, Zuccon G, Leelanupab T. Beyond GeneGPT: a multi-agent architecture with open-source LLMs for enhanced genomic question answering. arXiv preprint arXiv:251115061. 2025.

[33] Su H, Diao S, Lu X et al. ToolOrchestra: elevating intelligence via efficient model and tool orchestration. arXiv preprint arXiv:251121689. 2025.

[34] Pal A, Umapathi LK, Sankarasubbu M. MedMCQA: a large-scale multi-subject multi-choice dataset for medical domain question answering. In: Flores G, Chen GH, Pollard T, et al. (eds.), Proceedings of the Conference on Health, Inference, and Learning, pp. 248–60. PMLR, 2022.

[35] Bruce SJ . MMLU Medical Genetics Benchmark 2024. https://huggingface.co/datasets/shuyuej/MMLU-Medical-Genetics-Benchmark (5 November 2025, date last accessed).

[36] Hou W, Ji Z. GeneTuring tests GPT models in genomics. *BioRxiv* 2023;2023.

[37] Inc L . LangGraph: A Graph-Based Agentic Framework for LLM Applications 2024. Version 3.0.1, accessed: 05 November 2025. https://github.com/langchain-ai/langgraph.

[38] For Biotechnology Information NC . NCBI Resource Portal. Accessed: 05 November 2025. https://www.ncbi.nlm.nih.gov/.

[39] Hong G, Banos DT. Nano Bio-Agents (NBA): small Language Model Agents for Genomics. arXiv preprint arXiv:2509.19566. 2025.

[40] Abedini K, Silvello G. Biomedical Question Answering: Extending GeneGPT with the Code Act Paradigm. Master Thesis in Computer Engineering, University of Padova, 2025.

