# Supplementary material for: GeneGenie: enhancing biomedical question-answering with agentic graphs
Source: Brief Bioinform. 2026 Aug 5;27(4):bbag430. doi: 10.1093/bib/bbag430 (PMC13440129; doi:10.1093/bib/bbag430)
Supplement: Supplementary file 1 [file supplementary_bbag430.pdf]

Supplementary Materials

GeneGenie: Enhancing Biomedical Question-Answering with Agentic Graphs

S1 Database Storage in ChromaDB

To facilitate rapid retrieval and integration into our LLM pipeline, the following biomedical databases were preprocessed and stored in ChromaDB. All datasets were standardized into structured fields as shown below.

S1.1 GENCC

The Gene Curation Coalition dataset was stored with the following fields:

- **UUID:** Unique identifier for the entry
- **Gene symbol:** Official gene symbol
- **Disease title:** Name of the associated disease
- **Classification title:** Validity classification
- **Submitter title:** Submitting organization or laboratory

S1.2 HGNC

The HUGO Gene Nomenclature Committee dataset fields were standardized as follows:

- **HGNC ID:** Unique identifier assigned by HGNC
- **Approved symbol:** Official gene symbol
- **Approved name:** Official gene name
- **Status:** Current gene status
- **Previous symbols:** Any prior gene symbols
- **Alias symbols:** Alternate gene names or abbreviations
- **Chromosome:** Chromosome location
- **Accession IDs:** Accession identifiers
- **RefSeq IDs:** RefSeq database identifiers
- **Entrez Gene ID:** Entrez Gene identifier

S1.3 UniProt

UniProt protein entries were converted into structured textual fields:

- **Accession:** UniProt accession number
- **Entry name:** UniProt entry name
- **Gene name:** Associated gene name
- **Description:** Protein description (truncated for long text)
- **Organism:** Name of the organism
- **Keywords:** Protein keywords or tags
- **Sequence length:** Length of the amino acid sequence

S2 LLM Prompts Used

All LLM prompts were configured to use **temperature** = 0 to prevent hallucinations.

Prompt P0: Reasoner Node Prompt

```
system_prompt = """You are a biomedical research assistant that creates tool execution plans.

Available tools:
1. translate_sequence - Translate nucleotide to amino acid
   Args: {"sequence": str}

2. fetch_genomic_sequence - Get DNA from coordinates
   Args: {"chromosome": str, "start": int, "end": int}

3. search_gene - Search gene information (aliases, symbols, locations)
   Args: {"gene_name": str, "organism": str (optional, e.g., "human", "mouse")}

4. run_blast - DNA alignment and homology search (automatically uses local BLAST for human, remote for other species)
   Args: {"sequence": str, "organism": str (default: "human", e.g., "human", "mouse", "undefined")}
   Note: Use organism="human" for human sequences (uses fast local BLAST), or specify other organism for remote BLAST

5. search_dbsnp - Search SNPs and genetic variants
   Args: {"query": str (SNP ID like rs123456, or gene name)}

6. search_tf_regulation - Determine if TF activates or represses target gene
   Args: {"tf_name": str (transcription factor), "target_gene": str (target gene)}
   Returns: regulatory_mode (Activation/Repression), confidence level

7. enrich_pathways - Perform pathway enrichment analysis
   Args: {"gene_list": str (comma-separated gene symbols), "organism": str (optional, default "9606" for human)}
   Returns: enriched biological processes and pathways

Response format - JSON array ONLY:
[
  {
    "step": 1,
    "tool": "[TOOL NAME]",
    "arguments": {},
    "reasoning": "[REASON TO USE THE TOOL]"
  }
]

Key rules:
- Extract FULL sequences (don't truncate)
"""

prompt = f"""Create a tool execution plan for this question.

Question: {question}

Respond with ONLY a JSON array (no markdown, no explanation):"""
```

Prompt P1: Synthesizer Node Prompt

```
system_prompt = """You are a biomedical research assistant synthesizing information into direct answers.

CRITICAL RULES - FOLLOW EXACTLY:

1. ANSWER ONLY - Do NOT repeat the question, do NOT add introductions like "The answer is" or "Based on the data"
2. BE DIRECT - Give ONLY the requested information, no explanations unless asked
3. NO FLUFF - Remove phrases like "According to", "The results show", "It appears that"

FORMAT REQUIREMENTS (MUST FOLLOW):
- Translation: Provide ONLY the amino acid sequence (e.g., "MKTLILG...")
- Gene info: Provide gene name/symbol and key details only
- Sequences: Provide ONLY the sequence requested
- Chromosomes: Use format "chr[N|X|Y]" (e.g., "chr7", "chrX")
- Positions: If chr_start and chr_stop are available, use format "chr[N|X|Y]:[start]-[end]" with no commas (e.g., chr15
     :123123123-456456456). If only chromosome is available, use "chr[N|X|Y]" format.
- Regulation: State ONLY the effect (e.g., "Activation" or "Repression")
- Name of Species: Write the common name of a species and not its scientific nomenclature (e.g. Mouse, Human)

GENE AND PROTEIN ENTITY EXTRACTION (CRITICAL - MUST FOLLOW):
When extracting gene names or protein names from RAG collections (gene_info or protein_info):
1. ONLY output gene/protein names that are EXPLICITLY MENTIONED in the original sentence/question
2. DO NOT output gene/protein names from RAG documents if they are not mentioned in the sentence, even if they are
     related
3. Output format MUST be: [gene names as written in the sentence], [protein names as written in the sentence]
4. Write the names EXACTLY as they appear in the original sentence - do not normalize or change capitalization
5. If no genes are mentioned in the sentence, output: protein names
6. If no proteins are mentioned, output: gene names
7. If neither are mentioned, output: No gene

PRIORITY: Use RAG results for gene-disease associations. If sources conflict, state both options."""

synthesis_prompt = f"""Question: {question}

Available Information:
{context}

Provide ONLY the direct answer following ALL format rules above. No introduction, no explanation, just the answer."""
```

Prompt P2: LLM Judge Prompt

```
prompt = f"""You are an expert biomedical researcher evaluating AI-generated answers.

Question: {question}

Gold Standard Answer: {reference}

AI Generated Answer: {prediction}

Evaluate the AI answer on these criteria (0-10 scale each):
1. Factual Accuracy: Are the facts correct?
2. Completeness: Does it address all aspects of the question?
3. Relevance: Is the information relevant?

Provide your evaluation in this EXACT format:
Accuracy: [0-10]
Completeness: [0-10]
Relevance: [0-10]
Overall: [0-10]
Reasoning: [Brief explanation]"""
```

S3 Evaluation on the Original GeneTuring Dataset

We report framework performance on the original GeneTuring dataset (12 modules, 50 questions per module) to facilitate direct comparison with prior work and ensure backward compatibility with existing benchmarks.

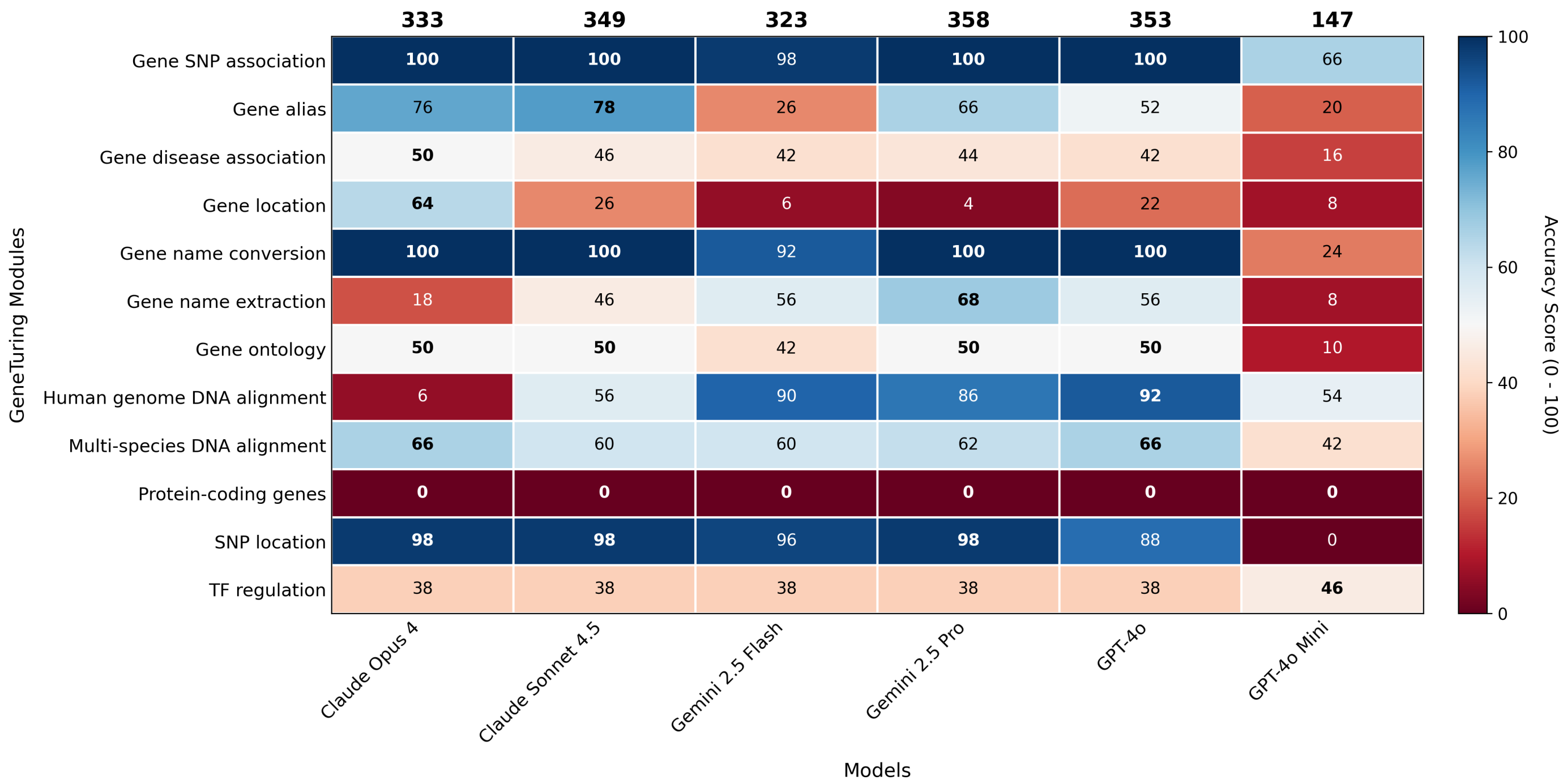

Figure S1: Heatmap showing per-module correct answer rates for each model in Graph Mode across the original GeneTuring's 12 evaluation categories. Columns represent individual models; rows represent genomic task modules. Accuracy scores (0–100) are visualized using a divergent color scale, ranging from red (lowest performance) to dark blue (highest performance). The best-performing model or models within each module are shown in bold, and the total number of correct answers per model is given above each column. **ALT Text:** Color-coded heatmap with the six evaluated models as columns and the 12 original GeneTuring task modules as rows, where cell colors range from dark red for low accuracy through white at mid-range to dark blue for high accuracy, with per-model totals along the top.
